# The Heineke-Mikulicz Principle for Hepaticojejunostomy Stricture

**DOI:** 10.1155/2012/454975

**Published:** 2012-08-05

**Authors:** Orhan Hayri Elbir, Kerem Karaman, Ali Surmelioglu, Erdal Birol Bostanci, Musa Akoglu

**Affiliations:** Department of Gastroenterological Surgery, Turkiye Yuksek Ihtisas Teaching and Research Hospital, 06410 Sihhiye, Ankara, Turkey

## Abstract

Benign anastomotic stricture after hepaticojejunostomy is one of the serious complications of biliary surgery. If left untreated, jaundice, cholangitis, or cirrhosis may develop. A 58-year-old male patient was admitted with benign hepaticojejunostomy stricture. The patient initially underwent an endoscopic retrograde cholangiography using double-balloon enteroscope, which was unsuccessful due to the sharp angle between the jejunal limb and the biliary tree. It was decided to perform surgery. During the operation, we performed Heineke-Mikulicz strictureplasty to the narrowed anastomosis. Patient's postoperative course was uneventful. At the end of followup, for 18 months, his liver enzymes were within normal ranges, and the ultrasound examination showed a patent hepaticojejunostomy anastomosis. The simplicity of the technique and the promising result support the applicability of the Heineke-Mikulicz principle in suitable cases as an alternative treatment approach for hepaticojejunostomy strictures.

## 1. Introduction

Benign anastomotic stricture after hepaticojejunostomy (HJ) is one of the serious complications of biliary surgery, which often presents with management difficulties. If left untreated, jaundice, cholangitis, or cirrhosis may develop. The incidence of anastomotic stricture following HJ has been reported as 4%–10% [1]. The potential risk factors for stricture formation are bile duct ischemia, multiple prior attempts at repair, intra-abdominal abscess or bile collection, external or internal biliary fistula, anastomosis in an undilated duct, preoperative and postoperative percutaneous biliary drainage, and patient comorbidities that might compromise visceral perfusion [2].

## 2. Case Presentation

A 58-year-old male patient was admitted with an acute attack of cholangitis due to HJ stricture. Three years earlier, he had undergone a pylorus preserving pancreaticoduodenectomy for a pancreatic mass. The diagnosis of the pancreatic mass was lymphoepithelial cyst.

**Figure 1:**
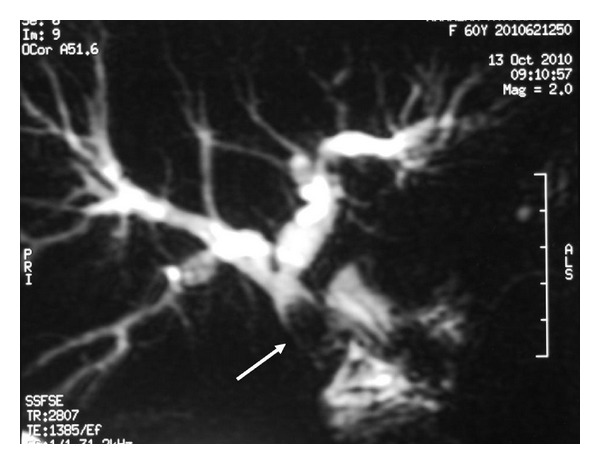
Magnetic resonance cholangiopancreatography revealed stricture of the hepaticojejunostomy anastomosis and mild dilatation of the biliary tree. The arrow shows the gallstone, which was localized in the common hepatic duct, just above the hepaticojejunostomy anastomosis.

**Figure 2:**
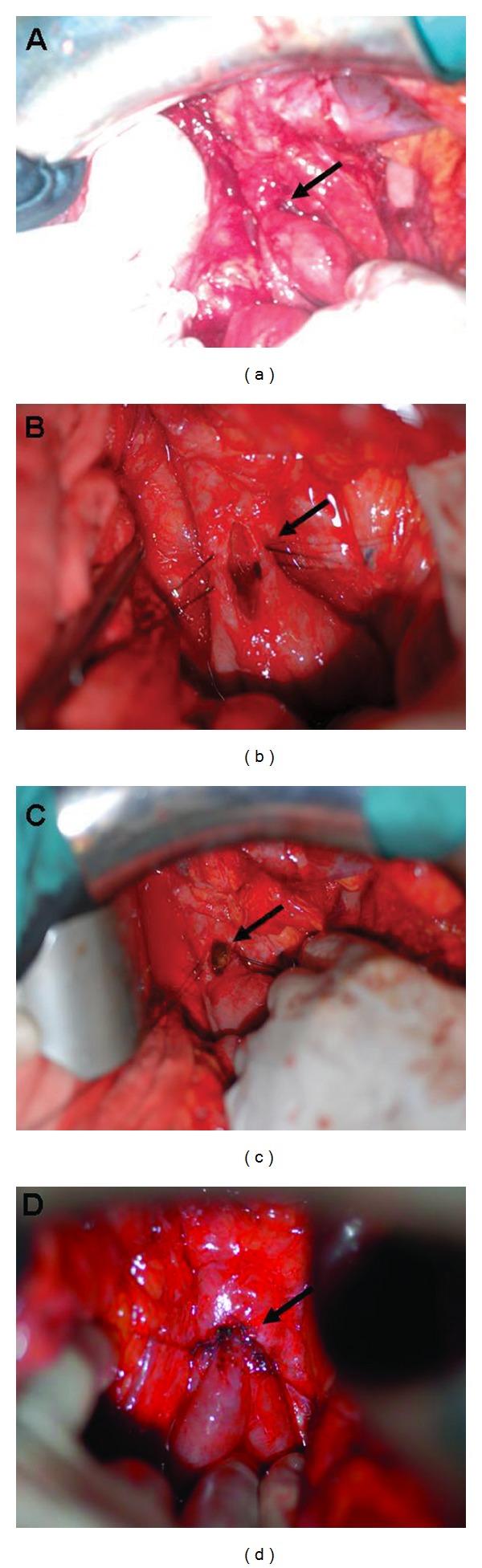
(a) The arrow shows the stricture of the hepaticojejunostomy anastomosis. (b) The arrow shows the vertical incision at the anastomotic line. (c) The arrow shows the gallstone, which was extracted trough the incision. (d) The arrow shows transverse closure of the vertical incision.

 At admission, blood chemistry tests showed slightly increased liver enzymes. The ultrasound examination revealed mild dilatation of the intrahepatic biliary ducts, which did not allow a percutaneous transhepatic cholangiographic intervention for balloon dilatation of the structured segment or biliary stenting. The magnetic resonance cholangiopancreatography (MRCP) detected stricture at the HJ anastomosis and a gallstone in the common hepatic duct just above the bilioenteric anastomosis (Figure 1). The patient underwent an endoscopic retrograde cholangiography using double-balloon enteroscope, which was unsuccessful due to the sharp angle between the jejunal limb and the biliary tree. Thus, it was decided to perform surgery. During exploration, the HJ anastomosis was found to be narrowed and covered with a scattered fibrotic tissue, with an outer diameter of less than 7 mm and 2 to 3 mm length. In addition, a stone was palpated in the common hepatic duct just above the HJ anastomosis. The length of the remnant common hepatic duct from the hilar region to the HJ anastomosis was approximately 1.5 cm. We decided to perform a Heineke-Mikulicz strictureplasty. A vertical incision of 2 cm was made to the anastomotic line. After the stone was extracted, the incision was resutured in a transverse fashion with simple suture technique using absorbable 4/0 polyglactin interrupted sutures, 2-3 mm apart. The strictureplasty provided an anastomotic patency of more than 1.5 cm (Figure 2). The patient's postoperative course was uneventful, and he was discharged on the fourteenth postoperative day after the duration of the antibiotic treatment for cholangitis was completed. At the end of followup, for 18 months, patient's liver enzymes and bilirubin levels were within normal ranges, and the ultrasound examination showed a patent HJ anastomosis.

## 3. Discussion

Treatment of benign biliary strictures consists of nonsurgical and surgical methods. Nonsurgical techniques are applied by endoscopic or percutaneous route using balloon dilatation or stent placement. Surgical methods vary from redo HJ to a number of special techniques such as biliary access loops (gastric, duodenal, or hepatocutaneous jejunostomy) to facilitate endoscopic intervention, purse-string anastomosis with an intra-anastomotic biogradable biliary stent placement [2, 3]. Redo HJ is the most common and preferred surgical method. Surgery or endoscopic biliary stenting are equally successful. However, when endoscopy is not successful, surgery is still feasible, but the reverse sequence is difficult when no access loop is available.

The Heineke-Mikulicz strictureplasty of longitudinal incision and transverse closure is a well-known concept in surgery. This principle has been extensively used for the correction of pyloric stenosis or to enlarge the diameter of narrowed small bowel segments mostly due to Crohn's disease [4]. Furthermore, it has also been successfully applied in urethral strictures [5].

One advantage of this technique is its simplicity, which shortens the operative time and eliminates the need of an anastomotic removal for redo HJ. However, it should be considered that this principle could not be applied in all HJ strictures, particularly in cases with a long and thin strictured segment. Secondly, a sufficient length of the remnant common hepatic duct (≥1 cm) is required for making the vertical incision with subsequent transverse closure. Further, the patency of the new HJ anastomosis must be at least larger than 1 cm to avoid recurrence.

Our case had no history of bile duct repair at the pancreaticoduodenectomy operation and his HJ stricture has been thought to occur due to compromised vascular supply after the HJ. A percutaneous transhepatic cholangiographic intervention for biliary stenting was initially planned but could not be applied due to mild dilatation of the biliary tree. The endoscopic route using double-balloon enteroscope was also unsuccessful because of the sharp angle between the jejunal limb and the biliary tree, which did not allow us to move the endoscope forward. Thus, a surgical intervention to redo HJ was planned. But during the operation we decided to perform Heineke-Mikulicz strictureplasty. To our knowledge, and after searching the English literature, our patient is the first published case to undergo Heineke-Mikulicz strictureplasty for HJ stricture. A stent could have been inserted through the strictureplasty anastomosis, as an option to facilitate biliary drainage. However, we did not prefer a stent because we found the patency of the strictureplasty adequate, and it should not also be forgotten that stent placement carries the risk of biliary infections such as ascending cholangitis. We do not expect restenosis because of the large patency provided by strictureplasty, and followup for 18 months showed no evidence of recurrence.

One disadvantage of strictureplasty is that of performing transverse closure on the fibrotic tissue, which may have the risk for anastomotic leakage or development of restricture formation. However, these risk factors are also valid for redo HJ.

In conclusion, the simplicity of the technique and the promising result support the applicability of the Heineke-Mikulicz principle in suitable cases—particularly in those where intervention techniques (percutaneous transhepatic biliary dilatation or stenting) cannot be applied for technical reasons—as an alternative treatment approach for HJ strictures. Further studies consisting of large patient populations are needed to reach a definite conclusion.
